# Gradual Reperfusion in Cardioplegia-Induced Cardiac Arrest

**DOI:** 10.3390/medicina60101564

**Published:** 2024-09-24

**Authors:** Mascha von Zeppelin, Florian Hecker, Harald Keller, Jan Hlavicka, Thomas Walther, Anton Moritz, Mani Arsalan, Tomas Holubec

**Affiliations:** 1 Department of Cardiovascular Surgery, University Hospital Frankfurt and Johann Wolfgang Goethe University Frankfurt, 60596 Frankfurt, Germany; mascha.vonzeppelin@unimedizin-ffm.de (M.v.Z.); florian.hecker@unimedizin-ffm.de (F.H.); jan.hlavicka@unimedizin-ffm.de (J.H.); thomas.walther@herz-frankfurt.de (T.W.); moritzanton@web.de (A.M.); mani.arsalan@unimedizin-ffm.de (M.A.); 2 Department of Cardiovascular Surgery, Clinical Perfusion, University Hospital Frankfurt and Johann Wolfgang Goethe University Frankfurt, 60596 Frankfurt, Germany; hrpkeller@t-online.de; 3 Department of Cardiology and Angiology, Medical Clinic I, University Hospital of the Justus Liebig University, 35392 Giessen, Germany

**Keywords:** cardiac surgery, cardioplegia-induced cardiac arrest, ischemia-reperfusion injury, gradual reperfusion, hyperoxemia, normoxemia

## Abstract

*Background and Objectives*: The majority of cardiac surgical procedures are performed using cardiopulmonary bypass and cardioplegia-induced cardiac arrest. Cardiac arrest and reperfusion may lead to ischemia-reperfusion injury of the myocardium. The aim of this study was to investigate whether gradual reperfusion with a slow increase in oxygen partial pressure leads to a reduction in reperfusion injury. *Materials and Methods*: Fifty patients undergoing elective cardiac surgery were included in this prospective randomized study. Patients in the hyperoxemic (control) group received conventional reoxygenation (paO_2_ 250–300 mmHg). Patients in the normoxemic (study) group received gradual reoxygenation (1st-minute venous blood with paO_2_ 30–40 mmHg, 2nd-minute arterial blood with paO_2_ 100–150 mmHg). Periprocedural blood samples were taken serially, and markers of myocardial injury were analyzed. In addition, the influence of gradual reoxygenation on hemodynamics, inflammation, and the overall perioperative course was evaluated. *Results*: There was a trend toward higher CK levels in the hyperoxemia group without statistical significance; however, CK-MB and troponin T levels did not show any statistical difference between the two groups. Potassium concentrations in the coronary sinus were significantly higher in the hyperoxemia group at 3 and 8 min after opening of the aortic cross-clamp (6.88 ± 0.87 mmol/L vs. 6.30 ± 0.91 mmol/L and 5.87 ± 0.73 mmol/L vs. 5.43 ± 0.42 mmol/L, respectively; *p* = 0.03 and *p* = 0.02). All other measurements did not show a statistical difference between the two groups. *Conclusions*: The use of gradual reperfusion in cardiac surgery with cardiopulmonary bypass and cardiac arrest is safe. However, it does not reduce ischemia-reperfusion injury compared to standard hyperoxemic reperfusion.

## 1. Introduction

Although the role of interventional and minimally invasive surgical therapy in cardiac medicine is increasing, the use of cardiopulmonary bypass (CPB) with cardioplegic arrest still remains essential for many procedures. Because the heart is deprived of blood and therefore oxygen and nutrients during cardiac arrest, without a protective mechanism this would lead to irreparable damage to the myocardium in a very short time [1].

However, the use of CPB can also lead to some adverse effects such as reperfusion injury, inflammation, and bleeding complications, which can lead to various organ damage, culminating in multi-organ failure [2].

Both ischemia and the subsequent reperfusion can cause global damage to the myocardial tissue. The repeated administration of the cardioplegic solution and the opening of the aortic cross-clamp to release the perfusion leads to a sudden oversupply of oxygen to the previously ischemic myocardium and thus to oxygen-mediated reperfusion injury [3]. This consists of tissue damage, acidosis, and hyperkalemia, which in turn can lead to cardiac arrhythmias, low-cardiac-output syndrome, myocardial stunning, and perioperative myocardial infarction. All of these factors can contribute to both prolonged hospitalization and increased mortality [4].

How to reduce ischemic-reperfusion injury after CPB has been the subject of numerous studies. However, the potential benefit of a stepwise increase in the oxygen supply during the reperfusion period has not been adequately investigated. Traditionally, reperfusion is performed with high partial pressure of oxygen (paO_2_) or hyperoxemia.

The aim of this study was to demonstrate whether a gradual increase in oxygenation during cardioplegia and reperfusion before opening the aortic cross-clamp can reduce the adverse effects of CPB and cardioplegia-induced cardiac arrest.

## 2. Materials and Methods

### 2.1. Ethical Statement

The study was approved by the Institutional Ethics Committee (reference number: 125/12; date of approval: 20 September 2012), and informed consent was obtained from each patient.

### 2.2. Study Design: Inclusion, Exclusion and Discontinuation Criteria

Between March 2013 and June 2015, 50 adult patients only, aged > 50 years, scheduled for elective cardiac surgery with an expected aortic clamp time > 75 min were enrolled in this prospective randomized clinical trial.

Discontinuation criteria were defined as interruption of the surgical procedure and accumulation of adverse events, such as increased mortality in either group. Operations resulting in myocardial lesions, such as mitral valve surgery, left ventricular aneurysm, septal myectomy, and atrial ablation, were considered exclusion criteria because they could affect the values obtained. If it became apparent intraoperatively that the procedure needed to be extended to include any of the above, the patient was excluded from the study. Patients were randomized by lot into two groups: 27 patients in the hyperoxemia group and 23 patients in the normoxemia group.

### 2.3. Definition of Clinical and Laboratory Parameter Collection

Defined laboratory parameters were collected intraoperatively and immediately postoperatively at fixed time points [Table 1], including the ischemia markers creatine kinase (CK), muscle brain-type creatine kinase (CKMB), and troponin T. In addition, potassium, lactate, SO_2_, pH, base excess, interleukin 6, and hemoglobin levels were determined. These values were obtained from both the central venous catheter (CVC) and the coronary sinus catheter (CS).

Hemodynamic measurements were taken using a Swan–Ganz catheter to determine cardiac output, cardiac index, resistance, and mean arterial pressure, as well as central venous pressure and heart rate. The first measurement was taken after induction of anesthesia, before the skin incision, and the second measurement was taken during skin suturing, before the end of surgery.

### 2.4. Surgical Procedure

Cardiac surgery was performed in a standardized fashion. Coronary artery bypass grafting (CABG), aortic valve surgery, aortic aneurysm surgery, and combined procedures were included as eligible cardiac procedures. The latter were CABG procedures combined with any valve or aortic surgery. Surgical access was either a conventional median sternotomy or a partial upper sternotomy (J-cut in the 4th intercostal space). CPB was used in all patients. Arterial cannulation for CPB was performed either via the aorta, femoral artery, or subclavian artery. Venous cannulation was performed either via the right atrium using a two-stage cannula, bicaval, or femoral vein. After CPB was established, the aorta was clamped and antegrade blood cardioplegia was administered. Each patient received a coronary sinus cardioplegia catheter for retrograde administration of cardioplegia. After successful surgery, cardiac reperfusion was initiated followed by release of the aortic cross-clamp. After transesophageal echocardiography, the patient was weaned from CPB and the chest closed in a standard manner.

### 2.5. Definition of Two Different Types of Cardioplegia and Reperfusion

**Figure 1:**
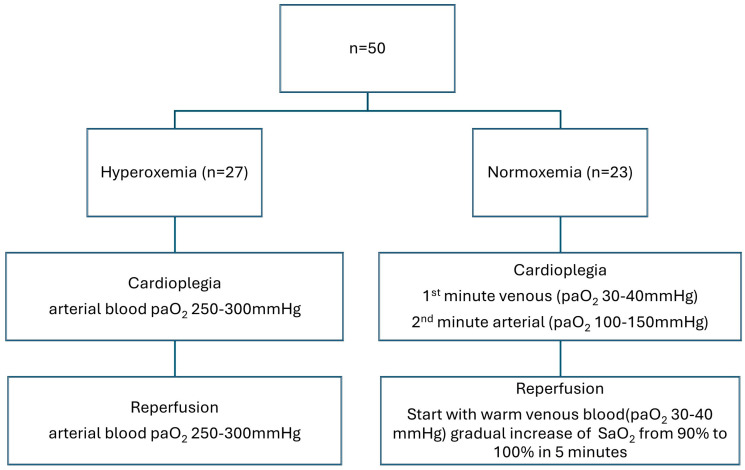
Study design.

The Frankfurt solution was used as the cardioplegic solution (modified Buckberg cardioplegia by adding glutamate/aspartate and reducing the potassium content in solution). The application rate was the same for both groups and was as follows: the first cardioplegia was administered at a flow of 300 mL/min, until cardiac arrest, followed by 200 mL/min for a total of 4 min. The target pressure in the aortic root was approximately 120 mmHg, or 80 mmHg after arrest. The second administration was given after 20 min with a flow of 200 mL/min for a total of 2 min. The third administration was congruent with the second administration. The flow always refers to the amount of blood/crystalloid cardioplegia in a ratio of 4:1. Reperfusion after opening the aortic cross-clamp was initially performed with warm venous blood (paO_2_ 30–40 mmHg), followed by gradual reoxygenation by increasing the systemic oxygen saturation (SaO_2_) from 90% to 100% during the first 5 min. This is the usual paO_2_ level in CPB. The study group differed from the routine (control) group in the administration of cardioplegia and reperfusion after opening the aortic cross-clamp [Figure 1].

Hyperoxemia group (control group): conventional hyperoxemic reoxygenation was performed during cardioplegia and during aortic cross-clamp opening. This means that the arterial blood had a paO_2_ of 250–300 mmHg.

Normoxemia group (study group): gradual reoxygenation was performed during the administration of cardioplegia and during the opening of the aortic cross-clamp. This was standardized as follows:

1st minute venous (paO_2_ 30–40 mmHg);
2nd minute arterial (paO_2_ 100–150 mmHg).

### 2.6. Laboratory Testing

Blood gas analyses were performed on point-of-care devices (ABL 800, BGA device, radiometer, Krefeld, Germany). Laboratory parameters (CK, CKMB, TropT) were determined in a standardized manner by the central laboratory of the University Hospital Frankfurt (Cobas 8100, Roche Diagnostics, Risch-Rotkreuz, Switzerland). The Swan–Ganz measurements (Carescape B850, GE Healthcare, Munich, Germany) were also performed according to a standardized procedure. The parameters were determined by the thermodilution method.

### 2.7. Statistical Analysis

Data were entered into a database using Microsoft Office Excel^®^ (version 2010 for Windows; Microsoft Corp, Redmond, WA, USA). Continuous variables are reported as the mean ± standard deviation or the median and range for non-normally distributed data. Categorical variables are reported using the number and percentage of observations. The Kolmogorov–Smirnov test was used to assess the normal distribution of data. The 2-sample t-test was used to compare continuous variables between the groups, assuming a normal distribution. If a non-normal distribution was assumed, the Wilcoxon–Mann–Whitney test was used. Pearson’s chi-squared test was used to compare categorical variables. A two-sided test was performed at an overall significance level of α = 5% (0.05). The *p*-values of the binary values were calculated using the four-field table. Statistical analysis was performed using Microsoft Office Excel^®^ (version 2010 for Windows; Microsoft Corp., Redmond, WA, USA) and BiAS© software (version 11.12, epsilon-Verlag GbR, Darmstadt, Germany, 1989–2020), where a *p*-value below 0.05 was considered statistically significant.

## 3. Results

### 3.1. Study Population

Of the total of 50 patients, 27 were in the hyperoxemia group (study group) and 23 in the normoxemia group (control group). Five patients had to be excluded after surgery, two from the hyperoxemia group and three from the normoxemia group. One patient underwent surgery without cardioplegia, two patients underwent myectomy. In one patient, an atrial myxoma was removed and, in another patient, a retrograde catheter could not be placed. The groups were evenly distributed based on their patient characteristics [Table 2]. There was no significant difference between the two groups.

### 3.2. Perioperative Early Clinical Data

In the hyperoxemia group, there were 13 CABG procedures: 2 valve-sparing aortic root replacements (David procedure); 5 combined aortic valve and CABG procedures; 1 combined aortic valve and replacement of the ascending aorta; 1 combined CABG and replacement of the ascending aorta; 1 combined David procedure and CABG; and 2 combined CABG, aortic valve, and replacement of the ascending aorta procedures were performed.

In the normoxemia group, there were seven CABG procedures, one replacement of the ascending aorta, two valve-sparing aortic root replacements (David procedure), two combined CABG and replacement of the ascending aorta, and eight combined CABG and aortic valve procedures were performed.

The CPB time was on average 23 min shorter in the normoxemia group compared to the hyperoxemia group (153.90 ± 29.4 min vs. 130.70 ± 44.97 min; *p* = 0.03). Aortic cross-clamp time showed no statistical difference between the two groups: normoxemia: median 35.75, IQR 78.5 vs. hyperoxemia: median 50, IQR 91 (*p* = 0.23). The mean length of hospital stay was 12 days, with a longer duration in the hyperoxemia group (15.44 ± 8.5) than in the normoxemia group (11.52 ± 5.06; *p* = 0.10), due to postoperative complications. There was no difference in the reinfarction rate between the two groups (0% hyperoxemia group vs. 5% normoxemia group [n = 1]; *p* = 0.9). The affected patient was successfully treated with coronary stenting. None of the patients experienced a neurological event such as a stroke. An organic cerebral psychosyndrome was observed in one subject in the hyperoxemia group. In addition to major cardiac and cerebrovascular complications, minor complications such as postoperative tachyarrhythmias (17.8%), respiratory insufficiency (15.55%), and postoperative delirium or decreased vigilance with a delayed wake-up reaction (20%) occurred. Pneumothorax or wound healing problems occurred in 4.4% of cases and pneumonia in 8.8%. Reasons for a prolonged normal ward stay were wound healing disorders, tachyarrhythmia, and pneumothorax. The average length of stay in the ICU was not statistically different between the two groups: median 1, IQR 4 (hyperoxemia) vs. median 1, IQR 2 (normoxemia) (*p* = 0.196). The main reasons for prolonged ICU stay were postoperative delirium, sepsis, or respiratory failure.

Three patients (6.6%) died during the hospital stay, two in the hyperoxemia group (8%) and one in the normoxemia group (4%) (*p* = 1.0). All deceased patients (n = 3) died due to sepsis with multi-organ failure. Five patients (11%) developed postoperative sepsis. One patient was in the normoxemia group, the other four were in the hyperoxemia group. Two patients had pneumogenic sepsis and three had an abdominal focus. Of these, one patient developed an abdominal compartment with consecutive reduced perfusion and peritonitis, while the others had small bowel and colonic ischemia.

### 3.3. Laboratory Parameters

There was a trend toward higher CK values in the hyperoxemia group compared with the normoxemia group, but there was no statistically significant difference between the two groups. The other main target values (CKMB i.S. and troponin T i.S.) did not differ between the two groups [Table 3].

In the CS and CVC blood gas analyses, only coronary sinus potassium concentrations were statistically significantly higher in the hyperoxemia group at 3 and 8 min (6.88 ± 0.87 mmol/L vs. 6.30 ± 0.91 mmol/L and 5.87 ± 0.73 mmol/L vs. 5.43 ± 0.42 mmol/L, respectively; *p* = 0.03 and *p* = 0.02) [Table 4]. However, the values after the 18 min blood sampling were no longer statistically different.

With regard to the hemodynamic parameters measured, the only statistically significant difference was found for the heart rate recorded at the beginning of the operation (*p* = 0.04), although this was no longer evident in later measurements [Table 5].

There was a statistical significance in the decreases in the interleukin 6 levels recorded preoperatively and immediately postoperatively after arrival in the ICU [Table 6]. This can be explained by the fact that some subjects already had a significantly elevated IL6 levels on admission to hospital, which also increased massively perioperatively. The values in the hyperoxemia group (11.232 ± 23.63 pg/mL vs. 5.96 ± 9.48 pg/mL; *p* = 0.04 and 1014.68 ± 1057.56 pg/mL vs. 477.08 ± 499.30 pg/mL; *p* = 0.02) were significantly higher than in the normoxemia group. This difference disappeared during the further postoperative course, so that after four hours postoperatively no significant difference could be detected.

## 4. Discussion

This study investigated the effect of gradual reperfusion after cardioplegic cardiac arrest during cardiac surgery in 50 patients. It was shown to be safe, and there were only a few differences compared with conventional reoxygenation. Many cardiac surgical procedures are still not possible without the use of a CPB and cardiac arrest. However, the reperfusion that follows the ischemia can cause global damage to the myocardial tissue [5]. This in turn leads to tissue damage, acidosis, and hyperkalemia. This can lead to cardiac arrhythmias, low cardiac output, myocardial stunning, and postoperative myocardial infarction [6,7]. As early as 1975, an animal study showed that normoxemia was associated with less end-organ damage [8]. In a human study, Abdel-Rahman et al. found that normoxemic reperfusion limits lipid peroxidation in the early reperfusion phase [5]. However, the study had only a small population of 19 patients. Abou-Arab et al. investigated whether hyperoxemic reperfusion could reduce postoperative complications, but found no effect on postoperative outcomes [9]. It should be emphasized that both studies included only patients with coronary artery disease. Our study has a heterogeneous cardio-surgical patient population.

Comparison of the few available studies on this topic is complicated by the fact that hyperoxemia and normoxemia were defined differently. Inoue et al. [10] reported paO_2_ values of 450–550 mmHg for their hyperoxemia group, whereas Abdel-Rahman et al. [5] reported values of above 250 mmHg. The values in the normoxemia group differ even more significantly. For example, in the study by Inoue et al. [10], normoxemia was considered to be 200–250 mmHg, whereas Abdel-Rahman et al. [5] only aimed for a paO_2_ of 50–70 mmHg. In the present study, hyperoxemia was defined as 250–300 mmHg, while normoxemia was defined as paO_2_ values of 30–150 mmHg. This means that the hyperoxemia of this study corresponds to the normoxemia of Inoue et al. [10]. With this in mind, it might be expected that a significant difference in the hyperoxemia group would only be seen at very high paO_2_ levels (e.g., Inoue et al. [10]). However, at lower paO_2_ levels (as in Abdel-Rahman et al. [5] and our study), no significant difference was found. All studies published to date, including this one, assume that the pre-diseased heart is already oxygen-deficient and that the rapid oxygenation in the context of hyperoxemia leads to the formation of oxygen radicals, which may induce reperfusion injury. Therefore, Karu et al. [11] investigated whether a pre-diseased heart could be preconditioned by preoxygenation. No direct reduction in reperfusion injury was found. Finally, it remains to be clarified whether the oxygen partial pressure during cardiac surgery influences patient outcomes. Possible effects, which include length of hospital stay, organ damage, stroke rate, and mortality, are admittable. In a study of 298 patients, McGuinness et al. [12] found no difference in ICU length of stay, total hospital length of stay, or 90-day survival as a function of oxygen saturation and oxygen partial pressure.

Brown et al. [13] conducted a study of 36 patients undergoing various cardio surgery procedures. They were randomized into a hyperoxemic group (paO_2_ 300–350 mmHg) and a normoxemic group (paO_2_ 150–250 mmHg). There was no significant difference in postoperative length of stay in the intensive care unit, which is consistent with the present study. The study differed from the present one in a number of key points: the oxygen partial pressure was higher in both groups, there was no gradual increase in paO_2_ in the normoxemic group, and patients with higher-grade renal failure, COPD, hypothyroidism, or with a preoperative neurological deficit (e.g., after stroke) were not included in the study. In addition, the anesthesia was different, with isoflurane used as a volatile anesthetic instead of total intravenous anesthesia.

### Study Limitations and Strengths

For some of the biomarkers studied, laboratory tests were not performed at all predefined time points. For example, at the end of the study, it was found that postoperative troponin was missing in many patients after 48 and 72 h due to different laboratory profiles between the ICU and normal ward. As a result, only a total of eight values ≥ 48 h were available for analysis in the hyperoxemia group, and only five values ≥48 h in the normoxemia group due to the shorter ICU stay. Therefore, the use of troponin at these two time points is limited. Furthermore, it was not recorded whether the total length of hospital stay was prolonged due to a preoperative delay. As a result, a postponed operation (e.g., due to another patient’s emergency operation) led to a longer total length of hospital stay. Due to the smaller numbers of patients in each group, despite the fact of randomization, some intra- and early postoperative data (CPB time, length of stay) differed based on more complex operations in the hyperoxemia group. This may have led to the lack of a significant difference in the total length of stay between the two groups in this study. However, as there was no significant difference in the length of hospital stay, it can be assumed that preoperative delays did not bias the analysis of the total length of stay. Swan–Ganz catheter measurements showed an improvement in cardiac index, cardiac output, and heart rate in both groups at the end of surgery. However, it should be noted that all patients received post-operative pacing, predominantly at a rate of 80 beats per minute. No data were collected on final postoperative intrinsic rhythm or cardiac output.

Finally, recruitment was difficult because many patients met the exclusion criteria preoperatively. These included surgically induced myocardial lesions, so patients with mitral valve disease or septal defects, for example, were not included in the study. However, the procedure had to be of a certain complexity in order to achieve an expected aortic clamping time of at least 75 min.

## 5. Conclusions

Gradual reperfusion after cardioplegic arrest during cardiac surgery is a safe procedure. There was no significant reduction in ischemia-reperfusion injury compared with classic hyperoxemia. A larger study may be needed to clarify the impact on clinical outcomes. Due to the lack of studies, there is still no recommendation from any professional society regarding the target paO_2_ values during cardiopulmonary bypass.

## Figures and Tables

**Table 1:** Timetable and overview of blood samples.

Time of Acceptance	Site of Sampling	Measurement
T1: Before induction of anesthesia	A. radialis	BGA
T2: 3 min after release of aortic cross-clamp	CVC	CK, CKMB, Troponin, BGA
	CS	BGA
T3: 8 min after release of aortic cross-clamp	CVC	CK, CKMB, Troponin, BGA
	CS	BGA
T4: 18 min after release of aortic cross-clamp	CVC	CK, CKMB, Troponin, BGA
	CS	BGA
T5: 33 min after release of aortic cross-clamp	CVC	CK, CKMB, Troponin, BGA
	CS	BGA
T6: 60 min after release of aortic cross-clamp	CVC	CK, CKMB, Troponin, BGA
T7: 4 h after release of aortic cross-clamp	CVC	CK, CKMB, Troponin, BGA
T8: 24 h after release of aortic cross-clamp	CVC	CK, CKMB, Troponin, BGA
T9: 48 h after release of aortic cross-clamp	CVC	CK, CKMB, Troponin, BGA
T10: 72 h after release of aortic cross-clamp	CVC	CK, CKMB, Troponin

CVC = central venous catheter, CS = coronary sinus; CK = creatine kinase, CKMB = creatine kinase of the muscle brain type, BGA = blood gas analysis.

**Table 2:** Pre-existing conditions and patient findings.

	Hyperoxemia (n = 25)	Normoxemia (n = 20)	*p*-Value
Age	68.36 ± 9.77 years	67.07 ± 8.55 years	0.64
EuroSCORE II	3.56 (3.225)	5 (3.65)	0.95
BMI	30.01 ± 5.77 kg/m^2^	27.93 ± 7.06 kg/m^2^	0.12
Arterial hypertension	20 (80%)	14 (70%)	0.66
TIA	2 (8%)	0 (0%)	0.57
Stroke	3 (12%)	2 (10%)	1.00
Atrial fibrillation	10 (40%)	3 (15%)	0.10
Diabetes mellitus	6 (24%)	7 (35%)	0.51
Peripheral arterial disease	7 (28%)	1 (5%)	0.10
COPD	3 (12%)	1 (5%)	0.76
Hyperlipidemia	10 (40%)	6 (30%)	0.91
Condition after myocardial infarction	9 (36%)	3 (15%)	0.21

BMI = body-mass Index; TIA= transient ischemic attack; COPD = chronic obstructive lung disease. EuroSCORE II presented median and interquartile range, and the rest presented as mean +/− SD or absolute (relative) frequencies.

**Table 3:** Creatine kinase, creatine kinase of the muscle brain type, and Troponin T in serum.

		Hyperoxemia	Normoxemia	*p*-Value
2 h	CK	394 (240) (n = 25)	418 (184) (n = 19)	0.312
	CKMB	41 (21) (n = 25)	37 (23) (n = 19)	0.922
	Trop T	522 (431) (n = 25)	483 (332) (n = 19)	0.24
4 h	CK	551.5 (823.25) (n = 24)	533 (341.5) (n = 19)	0.196
	CKMB	46 (60.25) (n = 24)	44 (27.5) (n = 19)	0.369
	Trop T	554 (624) (n = 23)	623 (862) (n = 16)	0.940
24 h	CK	714 (1314) (n = 25)	645.5 (315) (n = 20)	0.22
	CKMB	39 (33.5) (n = 23)	40 (23.25) (n = 20)	0.841
	Trop T	450 (397) (n = 15)	367.5 (441.25) (n = 12)	0.38
48 h	CK	684 (1231.5) (n = 24)	645.5 (362) (n = 20)	0.475
	CKMB	31.5 (28.75) (n = 22)	27.5 (15) (n = 18)	0.393
	Trop T	540 (204) (n = 6)	221.5 (210.5) (n = 4)	0.375
72 h	CK	744 (996) (n = 24)	330.5 (280) (n = 20)	0.146
	CKMB	22 (18) (n = 11)	21 (7.25) (n = 10)	0.625
	Trop T	-	-	-

CK and CKMB values in U/L, Trop T values in pg/mL; All data provided in median and interquartile range.

**Table 4:** Potassium concentration in the coronary sinus.

	Hyperoxemia	Normoxemia	*p*-Value
3 min	6.88 ± 0.87 (n = 23)	6.30 ± 0.91 (n = 20)	0.03
8 min	5.87 ± 0.73 (n = 25)	5.43 ± 0.42 (n = 20)	0.02
18 min	5.10 ± 0.49 (n = 24)	5.02 ± 0.33 (n = 20)	0.47
33 min	4.80 ± 0.5 (n = 22)	4.82 ± 0.36 (n = 14)	0.93

SD = standard deviation; values in mmol/L. All data presented as mean +/− SD.

**Table 5:** Swan–Ganz measurements 1 and 2.

		Hyperoxemia	Normoxemia	*p*-Value
CO	1st 2nd	3.76 ± 0.98 5.82 ± 1.45	3.70 ± 0.74 5.62 ± 1.67	0.90 0.53
HR	1st 2nd	52.70 ± 9.52 80.78 ± 14.35	59.19 ± 14.17 80.85 ± 12.85	0.04 0.57
MAD	1st 2nd	75.88 ± 13.32 69.15 ± 11.40	81.66 ± 11.63 72.40 ± 11.90	0.12 0.44
CVP	1st 2nd	10 (10) 8 (8)	11 (6.5) 9.5 (5)	0.306 0.44
PAM	1st 2nd	20 (6) 21.5 (5.5)	23 (12.25) 19.5 (9.5)	0.67 0.409
PAW	1st 2nd	12 (6) 12 (4.5)	11 (10) 10 (11.5)	0.821 0.766
CI	1st 2nd	1.80 ± 0.43 2.81 ± 0.64	1.91 ± 0.37 2.88 ± 0.77	0.34 0.80
SVRI	1st 2nd	3053 (1372) 1689.5 (712)	2907 (932.25) 1812 (694.75)	0.409 0.65

CO = cardiac output (L/min); HR = heart rate (beats/min); MAD = mean arterial pressure (mmHg); CVP = central venous pressure (cmH_2_O); PAM = pulmonary arterial mean pressure (mmHg); PAW = pulmonary arterial wedge (mmHg); CI = cardiac index (L/min/m2); SVR = systemic vascular resistance (dyn*s*cm^−5^); SVRI = systemic vascular resistance index (dyn*s*cm^−5^m^2^); SD = standard deviation. CVP, PAM, PAW, and SVRI presented in median and interquartile range, rest presented as mean +/− SD.

**Table 6:** Interleukin 6 from CVC.

	Hyperoxemia	Normoxemia	*p*-Value
Preoperative	5.1 (2.3) (n = 25)	3.1 (3.45) (n = 20)	0.097
Directly postoperative	656.4 (726) (n = 25)	303.9 (396.8) (n = 20)	0.044
4 h postoperative	497.9 (295.1) (n = 25)	332.25 (358.23) (n = 20)	0.245
1. POD	246.55 (132.55) (n = 24)	249.2 (200.45) (n = 20)	0.475
2. POD	206.5 (122.6) (n = 25)	138.2 (135.53) (n = 20)	0.812
Before discharge	23.9 (39.6) (n = 25)	30.8 (32.08) (n = 20)	0.596

SD = standard deviation, POD = postoperative day, values in pg/mL, all data presented as in median and interquartile range.

## Data Availability

Data supporting the reported results can be provided by the first author upon request.
